# Clinical impacts of genomic copy number gains at Xq28

**DOI:** 10.1038/hgv.2014.1

**Published:** 2014-07-24

**Authors:** Toshiyuki Yamamoto, Keiko Shimojima, Shino Shimada, Kenji Yokochi, Shinsaku Yoshitomi, Keiko Yanagihara, Katsumi Imai, Nobuhiko Okamoto

**Affiliations:** 1 Tokyo Women's Medical University Institute for Integrated Medical Sciences, Tokyo, Japan; 2 Department of Pediatrics, Tokyo Women's Medical University, Tokyo, Japan; 3 Department of Pediatric Neurology, Mikatahara General Hospital, Hamamatsu, Japan; 4 National Epilepsy Center, Shizuoka Institute of Epilepsy and Neurological Disorders, Shizuoka, Japan; 5 Department of Pediatric Neurology, Osaka Medical Center and Research Institute for Maternal and Child Health, Izumi, Japan; 6 Department of Medical Genetics, Osaka Medical Center and Research Institute for Maternal and Child Health, Izumi, Japan.

## Abstract

Duplications of the Xq28 region are the most frequent chromosomal aberrations observed in patients with intellectual disability (ID), especially in males. These duplications occur by variable mechanisms, including interstitial duplications mediated by segmental duplications in this region and terminal duplications (functional disomy) derived from translocation with other chromosomes. The most commonly duplicated region includes methyl CpG-binding protein 2 gene (*MECP2*), which has a minimal duplicated size of 0.2 Mb. Patients with *MECP2* duplications show severe ID, intractable seizures and recurrent infections. Duplications in the telomeric neighboring regions, which include GDP dissociation inhibitor 1 gene (*GDI1*) and ras-associated protein RAB39B gene (*RAB39B*), are independently associated with ID, and many segmental duplications located in this region could mediate these frequently observed interstitial duplications. In addition, large duplications, including *MECP2* and *GDI1*, induce hypoplasia of the corpus callosum. Abnormalities observed in the white matter, revealed by brain magnetic resonance imaging, are a common finding in patients with *MECP2* duplications. As primary sequence analysis cannot be used to determine the region responsible for chromosomal duplication syndrome, finding this region relies on the collection of genotype–phenotype data from patients.

## Introduction

Widespread application of chromosomal microarray testing has identified many new, contiguous gene syndromes.^1^ The accumulation of genotype and phenotype data of patients has narrowed the chromosomal regions responsible for common phenotypic features, and detailed examinations have isolated the genes responsible for certain human disorders. In particular, many X-chromosomal regions have been analyzed to investigate their relationship with X-linked intellectual disability (ID). For example, Froyen *et al.*^2^ reported a *de novo* microdeletion of Xp11.4 in which the calcium/calmodulin-dependent serine protein kinase (MAGUK family) gene (*CASK*; MIM #300172) is located. Najm *et al.*^3^ identified two additional deletions involving *CASK* in patients with mental retardation and microcephaly with pontine and cerebellar hypoplasia (MICPCH; MIM #300749). Furthermore, by screening 46 individuals with MICPCH, these authors identified two nucleotide alterations. These data suggest that *CASK* is involved in the pathogenesis of MICPCH, and mutations of this gene suggest that *CASK* is clinically significant in X-linked human disorders.^4^ Mutations in the Cdc42 guanine nucleotide exchange factor (GEF) 9 gene (*ARHGEF9*; MIM #300429) have been identified using the same strategy. Shimojima *et al.*^5^ identified a small deletion on Xq11.2 in patients with severe ID and epilepsy. Among the three deleted genes, *ARHGEF9* was of particular interest, and subsequent screening for nucleotide variations identified a nonsense mutation in this gene in 23 male patients with similar manifestations. Thus, it is now recognized that this gene is involved in X-linked ID and epilepsy (early infantile epileptic encephalopathy 8 (EIEE8); MIM #300607). This two-step approach, which involves narrowing down the chromosomal region followed by nucleotide screening, has identified multiple genes involved in human disorders. Currently, the use of next-generation sequencing has accelerated the identification of such genes.

This strategy has been applied to genes involved in pathogenesis in cases of haploinsufficiency because it is easy to confirm the involvement of genes in a phenotype when loss-of-function mutations are identified. In comparison, this strategy cannot be applied to gene mutations with dominant negative (gain-of-function) effects or to genes associated with pathogenesis in cases of copy number gain. For example, one of the gene regions that this strategy cannot be applied to is the Down syndrome-critical region because the genes responsible for Down syndrome cannot be identified through screening of nucleotide sequences.^6,7^ The isolation of genes or gene regions responsible for human disorders associated with gene-dosage-gain requires the collection of overlapping genotype and phenotype data from patients. Experimental animals would also be helpful to confirm the biological effects of the gene-dosage-gains.^8^

The most commonly reported chromosomal regions with copy number gains are Xq28 regions involving the methyl CpG-binding protein 2 gene (*MECP2*; MIM #300005).^9^ Indeed, we have previously reported seven patients with Xq28 duplications.^10,11^ In this review, we will discuss recent advances in the research on Xq28 genes that are responsible for ID when duplicated.

## *MECP2* Duplication

Since its first description,^12^ Rett syndrome (MIM #312750) has been known as a female-specific neurological disorder characterized by developmental regression, characteristic hand movements, autistic features and post-natal microcephaly.^13^ In 1999, the *MECP2* gene was shown to be responsible for Rett syndrome.^14^ Multiple pathogenic mutations have been identified in patients with Rett syndrome,^15^ and *de novo MECP2* mutations have been observed in females with typical manifestations of this syndrome.^16^ This finding indicates a dominant X-linked trait and lethality in hemizygous males^17^ and excludes aneuploidy of the X-chromosome in males (e.g., 47,XXY males)^18,19^ and specific *MECP2* nucleotide changes that lead to milder affects in females.^20^

**Figure 1:**
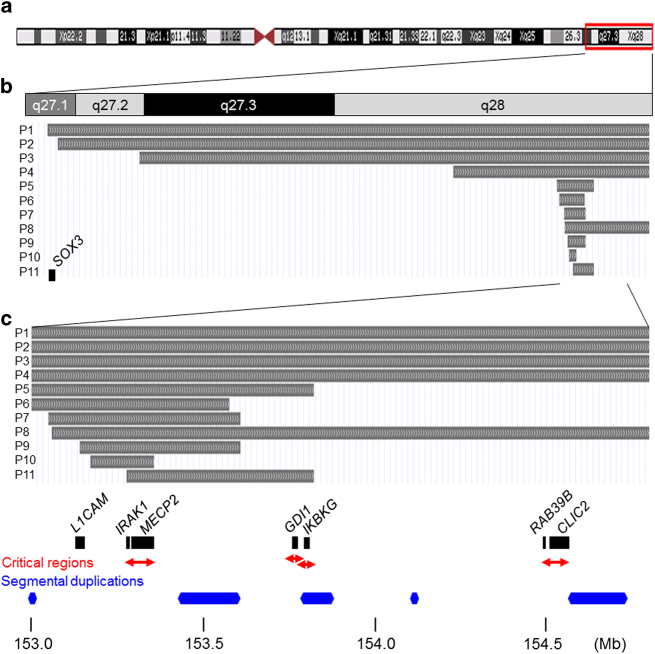
The genome map around Xq28. (**a**) A scheme of X chromosome downloaded from the UCSC genome browser. (**b**) Duplication regions identified in 11 patients are integrated by custom track and shown by grey bars. (**c**) Xq28 region is expanded. Examples of relevant genes are shown by black rectangles. Critical regions for distinct clinical features and segmental duplication regions are shown by red arrows and blue hexagons.

Conversely, *MECP2* duplication syndrome is a male-specific disorder associated with severe ID, intractable seizures and recurrent infections that lead to early death.^21^ This clinical condition was first recognized as Lubs-type X-linked mental retardation syndrome (MIM #300260),^22^ and its genetic etiology was identified as chromosomal duplications in the *MECP2* region.^17,23^ Varying sizes of the *MECP2* duplications (0.2–4.0 Mb) have been identified.^24–26^ Overlapping duplications narrowed down the shortest region overlapped in which *MECP2* and interleukin-1 receptor-associated kinase 1 gene (*IRAK1*; MIM #300283) were included (Figure 1).^27^ A twofold increase in *MECP2* expression was identified in patients compared with normal controls.^23^ Thus, *MECP2*, but no other contiguous genes in the duplicated region, is thought to be responsible for the neurological features of this condition.^27^ *MECP2* duplication syndrome is inherited as an X-linked recessive trait. Nearly all mothers of *MECP2* duplication patients are carriers of the duplication but do not develop severe ID due to skewed X-chromosome inactivation (XCI). Heterozygous females with random XCI, but not females with functional Xq28 disomy derived from translocation between autosomal chromosomes, show clinical manifestations of *MECP2* duplication syndrome but with a milder phenotype.^10,11^ As Ramocki *et al.*^27^ suggested that the majority of female carriers display neuropsychiatric symptoms before the birth of an affected son, careful follow-up of the families is required.

Although *MECP2* is related to genetic causes, there is a discrepancy in the XCI statuses of female patients carrying *MECP2* mutations (Rett syndrome) and those with *MECP2* duplications. Nearly all female patients with Rett syndrome show *de novo* mutations of *MECP2* and do not show skewed XCI. Conversely, *MECP2* duplication syndrome is associated with the X-linked recessive trait and skewed XCI in female carriers, which suggests that overexpression of neighboring genes in the duplicated region, rather than *MECP2* itself, may induce negative selection in the early embryo, leading to a preferential XCI.^28^ Alternatively, embryonic damage may be more severe in cases of *MECP2* duplication compared with *MECP2* nucleotide changes, although this remains controversial.

## *GDI1* Duplications

The GDP dissociation inhibitor 1 gene (*GDI1*; MIM #300104) is located on the telomeric neighboring region of the shortest region overlapped of *MECP2* duplication syndrome (Figure 1) and was identified as a gene responsible for ID because it was mutated in male patients.^29^ Heterozygous females manifested milder phenotypes, indicating an X-linked, semidominant inheritance. In 2007, microduplications, including *GDI1*, were independently reported with *MECP2* duplication by Froyen *et al.*^2^ and Madrigal *et al.*^30^ Vandewalle *et al.*^31^ reported the clinical entity associated with microduplications, including *GDI1* as a new duplication syndrome, and concluded that increased levels of *GDI1* are related to ID. The more increased copy number of *GDI1* correlated with more severe clinical phenotypes, including a Dandy–Walker malformation.^31^

## *IKBKG* Duplications

The kappa light polypeptide gene enhancer in B cells, inhibitor kinase gamma (*IKBKG*; MIM #300248), is located in the telomeric neighboring region of *GDI1* (Figure 1). Mutations in this gene lead to incontinentia pigmenti.^32^ A variety of distinct syndromes, including immunodeficiency with or without hypohidrotic ectodermal dysplasia, osteopetrosis, and lymphedema, are allelic disorders.^33^ Van Asbeck *et al.*^34^ reported a female patient with a *de novo* duplication of the *IKBKG* region in which *GDI1* was not included. This patient showed progressive macrocephaly, recurrent infections, ectodermal dysplasia, among others, but no ID. The XCI pattern was random in this patient.

## *RAB39B* Duplications

Giannandrea *et al.*^35^ identified mutations in the ras-associated protein RAB39B gene (*RAB39B*; MIM #300774) in patients with X-linked mental retardation associated with autism, epilepsy and macrocephaly. El-Hattab *et al.*^36^ identified recurrent microduplications involving *RAB39B* in male patients with cognitive impairment and behavioral abnormalities, including hyperactivity and aggressiveness. The duplication sizes observed in four unrelated patients were consistent at approximately 0.5 Mb and surrounded by segmental duplications (Figure 1). In each case, the mother was a non-symptomatic carrier with a skewed XCI. Vanmarsenille *et al.*^37^ identified a twofold increase in the expression level of *RAB39B* in the lymphocytes of patients compared with controls and confirmed decreased neuronal branching in Rab39b overexpressing mice. Andersen *et al.*^38^ reported a family with ID associated with an unbalanced inversion between Xp and Xq that resulted in duplication of the terminal region of Xq28. The duplicated region did not include *MECP2* but included *RAB39B* and the chloride intracellular channel 2 gene (*CLIC2*; MIM #300138). These results suggest that *RAB39B* may be responsible for the microduplication syndrome involving this region.

## Incidence of Xq28 Duplication

Currently, we have performed microarray-based comparative genomic hybridization analysis on 1250 patients with ID with or without other manifestations, such as congenital malformations, epilepsy or autistic features, in accordance with the previously described method.^7^ Two hundred thirteen patients showed one of the pathogenic genomic copy number aberrations (detection ratio of 17%). Among the identified aberrations, the most frequently observed copy number gain was the Xq28 duplication. In addition to the seven previously reported patients, four new patients presented Xq28 duplications, including *MECP2* (Table 1), resulting in a frequency of Xq28 duplications of 0.9%, which is comparable to the data reported by Lugtenberg *et al.*^39^ Furthermore, two of the patients were female.^10,11^

Patient 3 was a 4-year-old boy born at 37 weeks gestation and with a birth weight of 2268 g (10–50th centile). Generalized fetal edema secondary to severe anemia was noted. The patient had distinctive features, including synophrys, telecantus, flat nasal bridge, tented mouth, cleft palate, absent right thumb, bilateral radiohumeral synostosis and hypoplastic scrotum. This patient was diagnosed with Diamond–Blackfan anemia. Owing to these abnormalities, conventional chromosomal analysis was performed and showed 46,XY,add(3)(q27). Finally, microarray-based comparative genomic hybridization analysis and subsequent fluorescence *in situ* hybridization analyses confirmed an unbalanced translocation associated with functional disomy of Xq28. The deleted region (chr3: 197  052  877–198  022  430) included the ribosomal protein L35a gene (*RPL35A*; MIM #180468), which is responsible for Diamond–Blackfan anemia (patient no. 71).^40^ The developmental milestones were severely delayed in this patient: no neck control, no rolling over and no meaningful words. Neurological examination showed generalized hypotonia, and this patient never experienced epilepsy.

Patient 5 was a 7-year-old boy with a birth weight of 2,450 g (10–50th centile) at 37 weeks of gestation. Poor sucking due to generalized hypotonia was noted from early infancy. This patient had a history of multiple infections beginning from the first year of life. He began to suffer epileptic seizures from 1 year of age. Although he had been able to feed himself, he gradually lost the ability to eat from the age of 4 years. Thereafter, he was fed by tube, and a Nissen fundoplication was performed at the age of 5 years. At present, he is bedridden. He has never used meaningful words.

Patient 8 was aged 2 years and 9 months and had a birth weight of 2,422 g (3–10th centile), length of 46 cm (3–10th centile) and an occipito-frontal circumference of 32.5 cm (10–25th centile). His development was delayed, with head control and rolling over observed at 5 months, sitting alone at 13 months, standing with support at 24 months and walking with support at 33 months. He has made no eye contact and has exhibited intractable epilepsy since the first year of life.

Patient 11 was a 14-year-old male student at a school for disabled children. His height was 164.9 cm (50–75th centile), weight was 60.1 kg (75–90th centile) and occipito-frontal circumference was 60.0 cm (>97th centile), indicating macrocephaly. He has intractable epilepsy associated with myoclonic seizures and spasms. His gait is ataxic, there are no meaningful words, and he requires support during his daily life.

The Xq28 duplication patterns of the 11 patients from our laboratory were classified into two patterns, interstitial and terminal duplications, in 6 and 5 patients, respectively (Table 1). Interstitial duplications including *MECP2* were further classified into two types: a pure *MECP2* duplication in four patients and a *MECP2*+*GDI1* duplication in two patients. The terminal duplications were further classified into two types: functional Xq28 disomy derived from unbalanced translocations in four patients and pure terminal duplications (no translocation) in one patient (Table 1), which indicates the existence of variable patterns of Xq28 duplications. In cases of interstitial duplications, the distal ends overlap with segmental duplications (Figure 1), which indicates that these interstitial duplications are mediated by multiple segmental duplications in this region.^25^

**Figure 2:**
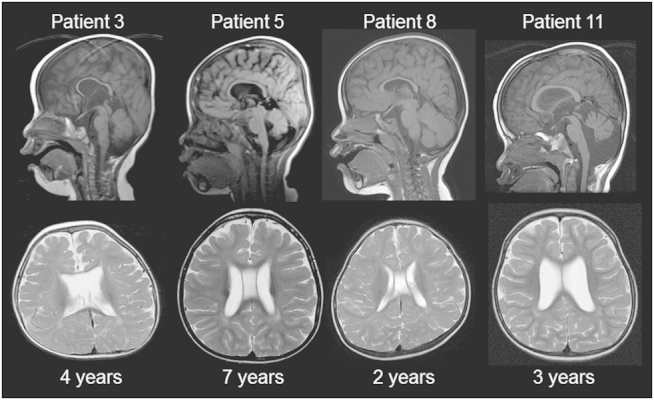
Brain magnetic resonance imaging (MRI) findings of new patients with Xq28 duplications. Sagittal T1 (up) and axial T2 (bottom)-weighted images are shown. All patients showed hypoplasia of the corpus callosum and T2 signal high intensities in the deep white matter. Three patients (other than patient 8) showed atrophies of the cerebellum and bilateral dilatations of the lateral ventricles, indicating age-dependent progression. Patient 3 showed a translucent septal defect, and patients 5 and 8 showed a verga cavity.

Previously, we reported abnormal findings in the white matter that were confirmed by brain magnetic resonance imaging) and suggested this as a common manifestation among patients with *MECP2* duplications.^10,11^ This finding was supported by those of Reardon *et al.*^41^ Four new patients with Xq28 also showed T2-weighted high intensity in the white matter (Figure 2). Dilatation of bilateral ventricles was also commonly observed.

## Genotype–Phenotype Correlation

The shortest region overlapped observed in patients with *MECP2* duplications associated with the typical phenotypic features of *MECP2* duplication syndrome included *MECP2* and the neighboring *IRAK1*, with a size of 0.2 M,^24^ which was supported by our study (Figure 1). Although Velinov *et al.*^42^ suggested that the distal Xq28 region beyond *MECP2* did not contribute to additional features, Honda *et al.*^43^ suggested that duplications of the *GDI1* region modified the clinical features in patients with *MECP2* duplications associated with hypoplasia of the corpus callosum. In this review, two new patients (patients 5 and 11) showed interstitial duplications beyond the shortest region overlapped of *MECP2* duplication syndrome that included the *GDI1* region. Careful observation of the patients’ magnetic resonance images showed hypoplasia of the corpus callosum, as suggested by Honda *et al.*,^43^ although there were no definite differences in the severity of clinical symptoms in patients with a *MECP2*+*GDI1* duplication compared with patients with pure *MECP2* duplications.

Patients with terminal duplication of Xq28 had significantly more severe neurological manifestations compared with patients with pure *MECP2* duplications. Four patients with interstitial duplications involving *MECP2*, but not *RAB39B*, could temporarily walk. However, patients with terminal duplications of Xq28 were immobile, which is a response derived from the integrated effects of the *RAB39B* region.

The maximum interstitial duplications observed in patients manifesting the phenotypic features of *MECP2* duplication syndrome involved a 4-Mb region.^26^ In comparison, some patients showed large terminal Xq duplications beyond Xq27,^44–47^ although most of the analyzed duplications had conventional G-banding levels. The largest duplication presented in this review was observed in patient 1, and a 139-Mb region in Xq27.1 was at the most proximal end. Furthermore, the neurological features in patient 1 were the most severe among the 11 patients studied because this patient showed severe hypotonia from early infancy and required continuous tube feeding. There were many episodes of life-threatening infection. In comparison with the second largest duplication that was observed in patient 2, the Y-box 3 gene (*SOX3*; MIM #313430) in the sex-determining region was located in the additional duplicated region in patient 1. This gene is related to X-linked ID and hypopituitarism,^48^ and duplication of this region is related to similar phenotypes, indicating dosage sensitivity.^49^ Therefore, the involvement of *SOX3* in the duplicated region could affect clinical severity in patients with Xq terminal duplications.

## Conclusions

The chromosomal patterns of Xq28 duplications and their clinical relevance have been discussed in this review. A review of the scientific evidence indicates that duplicated regions of the Xq28 chromosome that are responsible for ID can be separated into three distinct regions: *MECP2*, *GDI1* and *RAB39B*. Furthermore, *SOX3* may have modifier effects for severe ID. To establish the clinical significance of gene duplications, further analysis of genotype–phenotype data from these patients is required.

## Figures and Tables

**Table 1:** Summary of the aberration regions in the patients with Xq28 duplications

	*Age*	*Gender*	*Type*	*Region*	*Start* a			*Stop* a			*References*
					*Maximum*		*Minimum*	*Minimum*		*Maximum*	
Patient 1	3 years	M	Terminal; inverted X	*MECP2*+*GDI1*+*RAB39B*	139 433 084	//	139 531 883		155 270 560		Shimada *et al.*^10^ (Pt 3)
Patient 2	2 years	M	Terminal; t(X;Y)	*MECP2*+*GDI1*+*RAB39B*	139 743 195	//	139 801 223		155 270 560		Shimada *et al.*^11^ (Pt 2)
Patient 3	4 years	M	Terminal; t(3;X)	*MECP2*+*GDI1*+*RAB39B*	141 727 608	//	141 931 114		155 270 560		New
Patient 4	13 years	F	Terminal; t(12;X)	*MECP2*+*GDI1*+*RAB39B*	150 097 794	//	150 153 607		155 270 560		Shimada *et al.*^11^ (Pt 1)
Patient 5	7 years	M	Interstitial	*MECP2*+*GDI1*	152 819 509	//	152 857 869	153 822 717	//	153 822 717	New
Patient 6	5 years	F	Interstitial	*MECP2*	152 916 694	//	152 916 694	153 576 940	//	153 595 528	Shimada *et al.*^10^ (Pt 4)
Patient 7	20 years	M	Interstitial	*MECP2*	153 032 004	//	153 049 224	153 609 163	//	153 628 132	Shimada *et al.*^10^ (Pt 1)
Patient 8	2 years	M	Terminal; tandem	*MECP2*+*GDI1*+*RAB39B*	153 032 004	//	153 059 079		155 270 560		New
Patient 9	14 years	M	Interstitial	*MECP2*	153 083 345	//	153 140 483	153 609 163	//	153 628 132	Shimada *et al.*^10^ (Pt 2)
Patient 10	5 years	M	Interstitial	*MECP2*	153 140 483	//	153 177 776	153 357 772	//	153 406 233	Shimada *et al.*^10^ (Pt 3)
Patient 11	14 years	M	Interstitial	*MECP2*+*GDI1*	153 246 671	//	153 277 239	153 822 717	//	153 877 929	New

Abbreviations: F, female; GDI1, GDP dissociation inhibitor 1 gene; Interstitial, interstitial duplication; M, male; *MECP2,* methyl CpG-binding protein 2 gene; Pt, patient; t, translocation; Terminal, terminal duplication.

a Genomic positions refer to build19.
